# Parasegmental appendage allocation in annelids and arthropods and the homology of parapodia and arthropodia

**DOI:** 10.1186/1742-9994-5-17

**Published:** 2008-10-20

**Authors:** Nikola-Michael Prpic

**Affiliations:** 1 Georg-August-Universität Göttingen, Johann-Friedrich-Blumenbach Institut für Zoologie und Anthropologie, Abteilung für Entwicklungsbiologie, GZMB Ernst Caspari Haus, Justus-von-Liebig-Weg 11, 37077 Göttingen, Germany

## Abstract

The new animal phylogeny disrupts the traditional taxon Articulata (uniting arthropods and annelids) and thus calls into question the homology of the body segments and appendages in the two groups. Recent work in the annelid *Platynereis dumerilii *has shown that although the set of genes involved in body segmentation is similar in the two groups, the body units of annelids correspond to arthropod parasegments not segments. This challenges traditional ideas about the homology of "segmental" organs in annelids and arthropods, including their appendages. Here I use the expression of *engrailed*, *wingless *and *Distal-less *in the arthropod *Artemia franciscana *to identify the parasegment boundary and the appendage primordia. I show that the early body organization including the appendage primordia is parasegmental and thus identical to the annelid organization and by deriving the different adult appendages from a common ground plan I suggest that annelid and arthropod appendages are homologous structures despite their different positions in the adult animals. This also has implications for the new animal phylogeny, because it suggests that Urprotostomia was not only parasegmented but also had parasegmental appendages similar to extant annelids, and that limb-less forms in the Protostomia are derived from limb-bearing forms.

## Findings

Arthropods and annelids have their body divided into a series of repeated units that bear pairs of appendages in most cases. These body units and their appendages have long been regarded as homologous structures and have been the basis for uniting annelids and arthropods as sister taxa in the taxon Articulata [1]. The so-called "new animal phylogeny", however, does not support this close relationship between annelids and arthropods and rather places them in two different branches of protostome phylogeny termed Lophotrochozoa and Ecdysozoa, respectively [2]. This suggests that body segments and appendages in annelids and arthropods might have originated separately and are therefore not homologous. On the other hand recent results suggest that at least segmentation might have an ancient origin that predates or coincides with the origin of the Bilateria [3].

In arthropods the body units are first specified in a parasegmental register [4] and later these parasegments are transformed into segments by re-segmentation during embryonic development [5]. Intriguingly, recent work using the expression of the segmentation genes *engrailed *(*en*) and *wingless *(*wg*) has demonstrated that re-segmentation does not occur in annelids and the body units in annelids thus remain parasegments [6,7]. This also makes annelid appendages (parapodia) parasegmental rather than segmental structures and this is further evidenced by the expression of the appendage marker *Distal-less *(*Dll/Dlx*) [7]. This calls into question the homology between annelid parapodia and the appendages of arthropods (arthropodia). Here I show that in an arthropod species, the brine shrimp *Artemia franciscana*, the early body organisation including the limb primordia is identical to the annelid condition. This demonstrates that arthropodia like parapodia are initially parasegmental organs and suggests that both share a common evolutionary origin.

**Figure 1:**
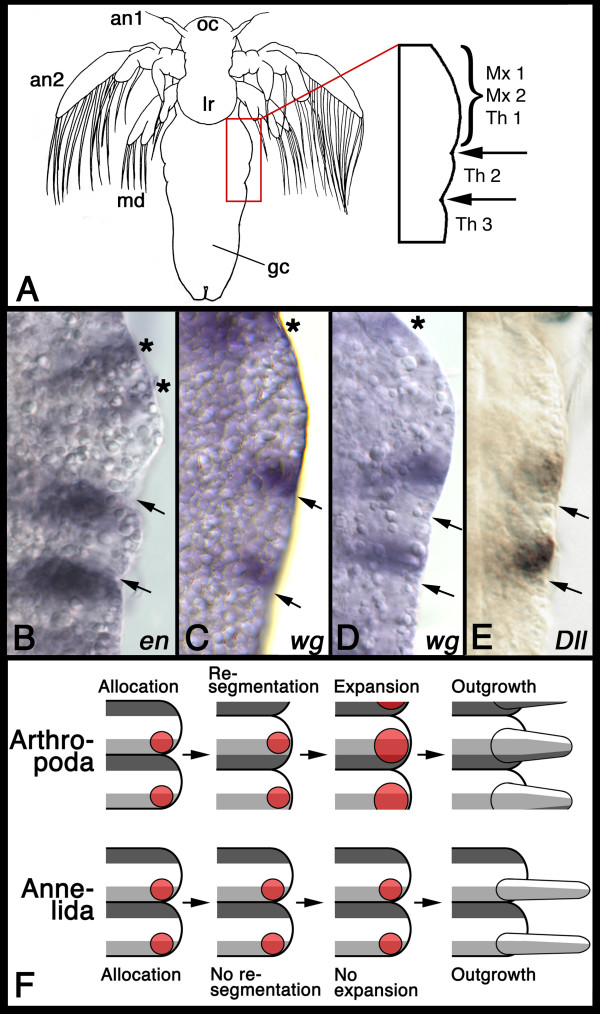
**Parasegments and limb primordia in the brine shrimp *Artemia franciscana***. (A) Explanatory drawing of a stage III nauplius larva. The anterior part consists of the ocular region (oc), labrum (lr), first antenna (an1), second antenna (an2) and mandible (md). The part of the trunk that is shown in B, C, and D is boxed in red and magnified. The trunk consist of a large bulge (containing the mesoderm for the presumptive segments of the first and second maxillae (Mx1, Mx2) and first thoracic appendages (Th1)) and a smaller bulge (containing the mesoderm for the second thoracic segment (Th2)). All following body units form from the growth cone. (B) Expression of *engrailed *in the anterior trunk. The asterisks denote the *engrailed *stripes of the future first and second maxillary segment. The *engrailed *stripes just posterior to the arrows are the stripes of the future first and second thoracic segment. The arrows in B-E point to the grooves between the parasegments. (C-D) Expression of *wingless *in the anterior trunk. The asterisk denotes the *wg *stripe of the first maxilla. The second maxilla does not (yet?) express *wg*. The nauplius in D is slightly older than the one in C. Note that the expression of *wg *is not directly anteriorly adjacent to the groove between large and small bulge in the older nauplius. (E) Expression of *Distal-less *in the anterior trunk reveals that the circle shaped appendage primordia are located anterior to the grooves. (F) Schematic summary of the model of appendage allocation in annelids and arthropods proposed here. Shown are two hemi-parasegments for each animal group, anterior is to the top. See text for details. Dark grey: *en *expression; light grey: *wg *expression; red: *Dll/Dlx *expression in the appendage primordia.

*Artemia franciscana *development includes a larval stage (nauplius) (Figure 1A). The nauplius consists of an anterior part, comprising the ocular region, labrum, first and second antenna and mandibles, and a posterior part, which is a more or less undifferentiated trunk. In nauplii at stage III (staging after [8]) this trunk region develops two bulges (boxed in red in Figure 1A) followed by a smooth region, the "growth cone". The reason for the two bulges is that there are already the mesodermal blocks forming beneath the ectoderm [8]. The large bulge contains, still in a single block, the mesoderm for several future segments (first and second maxillary, and first thoracic segment), the second smaller bulge contains the mesoderm for the second thoracic segment. These two bulges are thus the first morphological sign of subdivision in the trunk region. I used the expression of *en *[GenBank:X70939] and *wg *[EMBL:AM774593] to identify the location of parasegment boundaries in the trunk. *en *is expressed in the anterior portion of the smaller bulge and in the anterior part of the growth cone following the smaller bulge (Figure 1B). *wg *is expressed at the posterior border of the large bulge and in the posterior portion of the smaller bulge (Figure 1C). In some specimens, that are further into stage III, the *wg *stripe in the posterior part of the large bulge is separated from the morphological groove by about two cell diameters (Figure 1D). The significance of this is presently unclear. It could be a sign of the beginning resegmentation at this location or might be correlated with patterning mechanisms specific to the large bulge which is a complex structure comprising several future segments. Double-label in situ hybridizations of *wg *with *en *could clarify this, but have been technically impossible in *Artemia *so far. Based on the opposition of *wg *and *en *expression across the morphological grooves between large bulge and small bulge (at least in early stage III) and between small bulge and growth cone (throughout stage III) the morphological units at stage III are still parasegments and the morphologically visible indentations (grooves) between them coincide with the parasegment boundaries. I then used the expression of the appendage marker *Dll *[EMBL:AM774594] to identify the appendage primordia. *Dll *expression shows that the first appendage primordia are already specified at stage III (Figure 1E). Surprisingly, *Dll *is expressed in groups of cells anteriorly adjacent to the grooves and thus in front of the parasegment boundary. Thus, the limbs in *Artemia *initially are parasegmental structures, identical to the parapodia of the annelids (see [7]).

Hejnol and Scholtz [9] have studied limb primordium formation (as marked by Dll protein expression) in the crustaceans *Orchestia cavimana *and *Porcellio scaber*. Because in these species the cell lineage is known, these authors were able to demonstrate that the limb primordium starts as a single cell and expands by activation of Dll in adjacent cells. I propose that arthropods use this expansion mechanism to transform their initially parasegmental appendage primordia into segmental appendages after re-segmentation (Figure 1F, upper row). By contrast, annelids do not re-segment and the clonal boundaries of the parasegments are retained as the definite morphological borders between the body units. I suggest that because of this, the restrictive influence of the morphological borders between the parasegments cannot be overcome by the primordia of the parapodia, resulting in a lack of expansion of the primordia (Figure 1F, lower row). This model thus derives the adult arthropod condition from a common body organisation in immature arthropods and annelids by the arthropod-specific processes of re-segmentation and subsequent expansion of the appendage primordium across the parasegment boundary.

**Figure 2:**
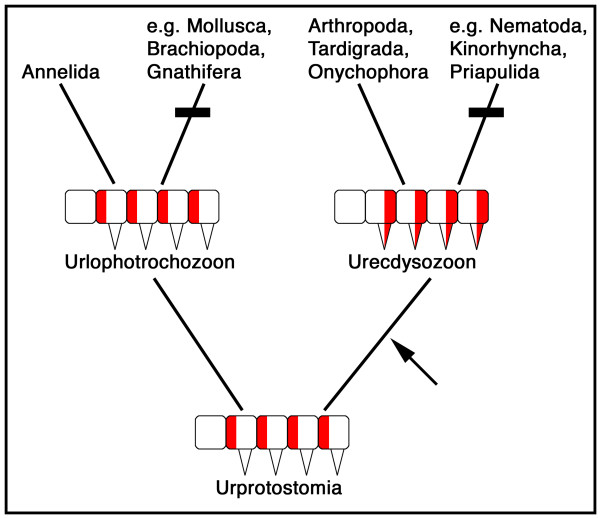
**Evolutionary hypothesis for the origin and loss of appendages in the Protostomia**. Based on the model shown in Fig. 1F, the common ancestor of Lophotrochozoa and Ecdysozoa, termed Urprotostomia [6], was parasegmented and had parasegmental appendages. No change of this ancestral condition is required in the lophotrochan lineage; the ancestral lophotrochozoan ("Urlophotrochozoon") is virtually identical in body organization to Urprotostomia. In the ecdysozoan lineage the processes of re-segmentation and appendage primordium expansion were evolved. This likely happened before the split of all extant ecdysozoans (arrow), based on the arthropod-like expression of *en *in the appendages of onychophorans [12]; the ancestral ecdysozoan ("Urecdysozoon") thus had already an adult body organization consisting of segments and segmental appendages. The limb-less forms in both Lophotrochozoa and Ecdysozoa must then be derived from limb-bearing forms by secondary loss of appendages (denoted by the black bars).

In the adult animals arthropodia and parapodia have different positions on the body (segmental versus parasegmental). This argues against their homology. The present data, however, show that the primordia of these appendages have identical positions in the immature animals and thus arguably derive from a common ancestral structure (i.e. they are homologous). This strongly suggests that Urprotostomia, the last common ancestor of annelids and arthropods, was not only already parasegmented [6,7], but also had parasegmental appendages. This also has consequences for the discussion of animal evolution [10,11] because it suggests, that the last common ancestor of the Lophotrochozoa (Urlophotrochozoon) and of the Ecdysozoa (Urecdysozoon) had appendages and the appendage-less forms in both Lophotrochozoa and Ecdysozoa have lost their appendages secondarily (Fig. 2). Data from the Onychophora show that in older embryonic stages *en *is expressed in the posterior portion of the appendages and thus in a segmental fashion like in arthropods [12]. This suggests that re-segmentation was already present in the Urecdysozoon (see arrow in Fig 2).

## Methods

### Artemia culture and fixation

*Artemia franciscana *cysts were purchased from Dohse Aquaristik (Grafschaft-Gelsdorf, Germany) and were activated in seawater (34 g seasalt per litre) with constant oxygen supply at 25°C. Larvae were harvested after 24, 48 or 72 hours. Fixation of nauplii proved to be difficult. Best results were achieved after washing the nauplii in DanKlorix cleaner (Colgate-Palmolive, Hamburg, Germany) for 5 minutes and subsequent fixation in PEMFAH (3 ml PEMS, 450 μl formaldehyde (37%), 5 ml heptane) at 4°C over night. After fixation the nauplii were transferred to methanol and stored at -20°C. Best possible tissue fixation was determined to be the case when after the methanol treatment the orange pigment of the nauplius eyes was preserved and the tissue within the appendages was white rather than clear.

### In situ hybridisation

*Artemia *nauplii were rehydrated stepwise in PBST and then sonicated for 5 seconds with a tip sonifier (Branson Cell Disruptor B15). Sonication was optimal when more than 50% of all nauplii were destroyed. They were then treated according to the published protocol for *Glomeris marginata *[13] with the following modifications: acetylation lasted for 1 hour; anti-Dig antibodies were preabsorbed against fixed and sonicated nauplii for 24 hours at 4°C in PBST supplemented with 2% sheep serum.

### Gene cloning

For RNA extraction, cysts were activated in sterilized and filtered sea water and the nauplii were harvested 48 hours later. Total RNA was extracted using the Trizol reagent (Invitrogen). cDNA was synthesized from this total RNA using the SuperScript II system (Invitrogen). The *Artemia engrailed *gene has been reported previously [14] and a fragment was cloned with gene specific primers designed on the basis of the published sequence [GenBank:X70939]. A fragment of the homeobox of the *Artemia Distal-less *gene has been cloned using the previously published primers eDP fw, eDP bw, iDP fw and iDP bw [13]. Within this sequence two nested gene specific primers were designed and were used in two subsequent PCR reactions together with the primer DP dlxm1 [13] to amplify a larger portion of the gene that spans from the homeobox to the DLX-1 motif [15]. A fragment of the *Artemia wingless *gene was cloned using the primers reported previously [4].

## Competing interests

The author declares he has no competing interests.
